# Homologous recombination-DNA damage response defects increase TMB and neoantigen load, but not effector T cell density and clonal diversity in pancreatic cancer

**DOI:** 10.1186/s40164-025-00673-0

**Published:** 2025-06-18

**Authors:** Mengyue Lei, Jessica Gai, Thomas J. McPhaul, Huijuan Luo, Penghui Lin, Dongbing Liu, Michael Pishvaian, Nicholas J. Roberts, Kui Wu, Jin He, Lei Zheng

**Affiliations:** 1 Guangdong Provincial Key Laboratory of Human Disease Genomics, Shenzhen Key Laboratory of Genomics, BGI Research, Shenzhen, 518083 China; 2 Institute of Intelligent Medical Research (IIMR), BGI Genomics, Shenzhen, 518083 China; 3 Sidney Kimmel Comprehensive Cancer Center, Johns Hopkins University School of Medicine, Baltimore, MD 21287 USA; 4 Department of Oncology, Johns Hopkins University School of Medicine, Baltimore, MD 21287 USA; 5 Pancreatic Cancer Precision Medicine Center of Excellence Program, Johns Hopkins University School of Medicine, Baltimore, MD 21287 USA; 6 Department of Surgery, Johns Hopkins University School of Medicine, Baltimore, MD 21287 USA; 7 Department of Pathology, Johns Hopkins University School of Medicine, Baltimore, MD 21287 USA; 8 Mays Cancer Center, University of Texas Health San Antonio, 7979 Wurzbach Road, MC8026, San Antonio, TX 78229 USA

**Keywords:** Pancreatic ductal adenocarcinoma (PDAC), Homologous recombination-DNA damage response defects, Tumor mutation burden (TMB), T cell infiltration, T cell clonal diversity, Immune checkpoint inhibitors

## Abstract

Pancreatic ductal adenocarcinoma (PDAC) is highly resistant to chemotherapy. However, PDAC with germline *BRCA* mutations, which lead to homologous recombination (HR) deficiency (HRD), demonstrated an increased sensitivity to platinum-based chemotherapy regimens. This increased chemosensitivity was also seen in PDACs with germline or somatic mutations in the DNA double-strand damage response (DDR) genes beyond canonical HR genes such as *BRCA1, BRCA2, and PALB2*. However, there are no consensus methods to determine HRD status; and neither is there a well-defined list of HR-DDR genes. In addition, how HRD and/or HR-DDR gene mutation status impacts the tumor immune microenvironment including tumor mutation burden (TMB), neoantigen load, T cell receptor (TCR) repertoire, and effector T cell infiltration is unknown. Thus, in this study, we developed a new method to categorize PDACs into HRD-positive and HRD-negative subgroups by using results from whole exome sequencing, whole genome sequencing, or both into consideration. We classified a cohort of 89 PDACs into HRD-positive (n = 18) and HRD-negative (n = 69) tumors. HR-DDR gene variants were identified more frequently in HRD-positive PDACs than HRD-negative PDACs, with *RAD51B*, *BRCA2* and *ATM* alterations most frequently identified in HRD-positive PDACs. Notably, TMB and neoantigen load was significantly higher in HRD-positive PDACs compared to HRD-negative tumors. Interestingly, HRD-positive PDACs, PDACs with high tumor mutational burden, and PDAC with high neoantigen load were all associated with lower CD8 + T lymphocyte infiltration and T cell clonal diversity, suggesting a mechanism of resistance to immune checkpoint inhibitors (ICIs). Therefore, this study suggests that treatments to enhance effector T cell infiltration and T cell clonal diversity may overcome resistance to ICI-based immunotherapy in HRD-positive PDACs.

**Supplementary Information:**

The online version contains supplementary material available at 10.1186/s40164-025-00673-0.

To the editor

BRCA1, BRCA2, and PALB2 are critical components of the homologous recombination (HR) machinery, patients with germline *BRCA1/2*-or *PALB2*-mutated Pancreatic ductal adenocarcinomas (PDACs) are categorized as having tumors with homologous recombination deficiency (HRD)(HRD-positive). PDACs with germline or somatic mutations in the DNA double-strand damage response (DDR) genes, similar to those with germline *BRCA* mutations have an increased sensitivity to platinum-based chemotherapy regimens [1]. To effectively target HRD-positive PDACs, a comprehensive evaluation of HR-DDR mutations and their associated HRD status is necessary. Thus, we performed an analysis of HR-DDR alterations in PDACs from 89 patients, comprised of 41 patients with surgically resectable tumors and 48 patients with locally advanced pancreatic cancer (LAPC) (Tables S1–4) [2, 3]. We developed a new, combined WES/WGS scoring system [4–10] to categorize PDAC specimens into HRD-positive and HRD-negative tumors. A specimen was considered HRD-positive if either the WES or WGS HRD score was > 2(HRD c1 or c2, respectively), or the WES HRD score was > 0 and the WGS HRD score > 1(HRD c3) (Fig S1). Only one patient was classified as both HRD c1 and HRD c2. Using our novel scoring method, 18 of 89 patients with PDAC had specimens that were classified as HRD-positive (20.2%), while 69 patients were had specimens that were classified as HRD-negative (79.8%) (Fig. 1A–C; Table S5). HRD-positive PDACs showed a trend towards a higher mutation number (p < 0.0001), CNV burden (p = 0.0004) and SV number (p < 0.0001) per tumor when compared to HRD-negative PDACs.

**Fig. 1:**
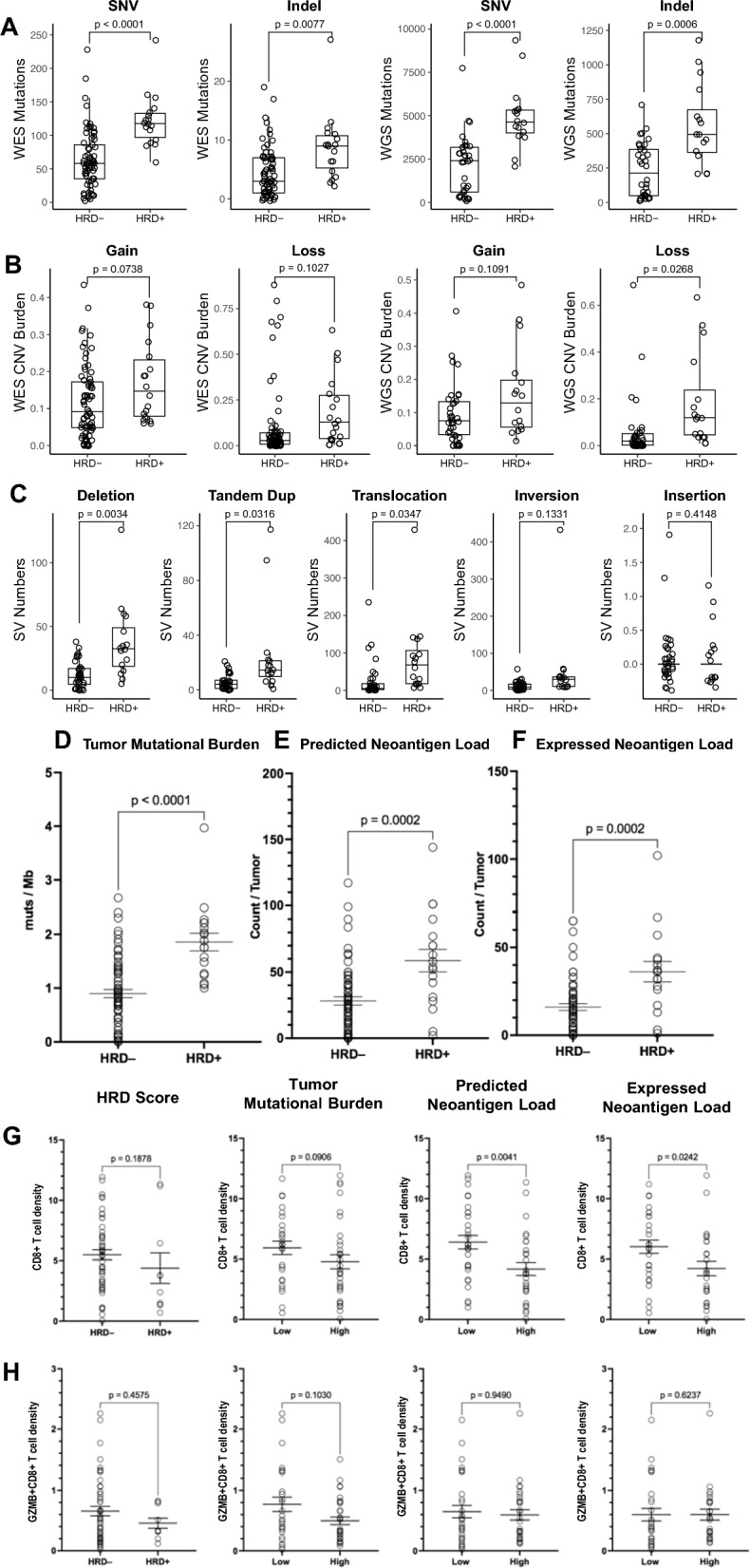
Genomics landscape of all PDAC samples and correlation between tumor mutational burden, neoantigen load, intratumoral effector T cell densities, and HRD classification. **A**. SNV or Indel mutation numbers per WES (left panels) and per WGS (right panels). **B.** CNV burden per WES (left panels) and per WGS (right panels), Gain: total copy number > 2; Loss: total copy number < 2. CNV burden is defined as CNV length divided by whole genome length for WGS and as CNV length divided by target region length for WES. **C.** Numbers of five different types of SV, including deletion, tandem dup (tandem duplication), translocation, inversion and insertion, as indicated, according to WGS. t test was used to compare between HRD-positive PDACs (n = 18) and HRD-negative PDACs (n = 69). Many CNV and SV parameters are significantly increased in HRD-positive PDACs. In this study, a new method was developed to categorize PDACs into HRD-positive and HRD-negative subgroups using both WES and WGS results. None of the current strategies of predicting HRD in PDACs is ideal; and functional assays to predict HRD status require further validation. Note that the scoring system developed and used in this study and may have missed the true HRD-positive tumors or have categorized non-HRD tumors into HRD-positive subgroup. In support of our scoring system, the tumors categorized as HRD-positive carried more HR-DDR gene alterations, particularly alterations resulting in loss-of-function. While most patients with HRD-positive PDAC did not have an identifiable, loss-of function alteration in a HR-DDR gene, the majority of the tumors classified as HRD-positive carried a VUS in a HR-DDR gene. An unknown number of these VUS may actually be pathogenic and the list of HR-DDR genes in this study is incomplete. Therefore, the scoring system developed and used to predict HRD-positive HRD used in this study would be valuable to select tumors for the functional assay development and validation. A tumor is considered to be HRD (HRD-positive) if the WES HRD score > 2 (as the HRD category 1; c1), if the WGS HRD score > 2 (as the HRD category 2; c2): or if the WES HRD score > 0 and the WGS HRD score > 1 (as the HRD category 3; c3) (Supplemental Fig. 1). We use those published tools and thresholds defined by each tool. Either WES or WGS score > 2 would mean that HRD can be positively detected by at least one tool using q strict threshold and a second tool using a loose threshold or HRD can be positively detected by at least three tools using a loose threshold. Either WES or WGS score > 2 would meet the criteria for HRD. For those samples with both WES and WGS, WES score > 0 and WGS score > 1 would mean that HRD is positively detected by at least one WES tool using a loose threshold and HRD is positively detected by at least two WGS tools using a loose threshold. Among the 18 patients with HRD-positive PDACs (Table S2), 1 was classified as both HRD c1 and HRD c2, 2 were HRD c1, 12 were HRD c2, and 3 were HRD c3. Using WES, we identified 2,190 somatic single-nucleotide variants (median 117.5 per sample, range 60–242 per sample),157 somatic insertion/deletion mutations (median 9 per sample, range 2–27 per sample), and CNV burden (median 0.37 per sample, range 0.1–0.7 per sample) in the HRD-positive PDACs. Using WGS, 3,234 genome-wide, somatic structural variants (SVs) (median 157 SVs per sample, range 41–617 per sample) were identified (Table S4). In HRD-negative PDACs, we identified 4,378 SNVs (median 58 per sample, range 2–228 per sample), 315 somatic indels (median 3 per sample, range 0–19 per sample), and CNV burden (median 0.16 per sample, range 0–0.88 per sample) using WES. While based on WGS, 1,596 genome-wide, somatic SVs (median 28.5 SVs per sample, range 0–195 per sample) were identified (Table S4). Tumor mutational burden **D**, predicted neoantigen load **E** and expressed neoantigen load **F** were compared between HRD-positive PDACs (n = 18) and HRD-negative PDACs (n = 69) by t tests. Predicted neoantigen load: all the neoantigens that were predicted by binding affinities. Expressed neoantigen load: predicted neoantigens that were expressed according to RNA-seq. The results showed that HRD-positive PDACs are associated with high TMBs and high neoantigen loads. PDACs were divided into “low” vs. “high” subgroups using the median values of Tumor mutational burden, predicted neoantigen load, and expressed neoantigen load, respectively, as classification cutoffs. Intratumoral CD8 + T cells **G** and GZMB + CD8 + T cells **H** were compared between subgroups by t test. Data shown as mean ± SEM. Comparison was made by t tests, with p values shown. Note that the association between higher neoantigen load and lower CD8 + T lymphocytes was statistically significant (p = 0.0041). Nevertheless, HRD status, TMB, and neoantigen load, were not correlated with tumor-infiltration of cytotoxic effector T lymphocytes (Granzyme B + CD8 + T cells). Thus, HRD-positive PDACs did not show increased tumor CD8 + T cell infiltration. To the contrary, CD8 + T cell density was decreased in HRD-positive PDACs compared to HRD-negative PDACs. Correlation between HRD status and expression of PD-L1 and LAG3, infiltration of T regulatory cell and myeloid derived suppressive cell (MDSC), and cytokine profiling will be investigated in the future studies

We identified genetic variants that were either clinically significant variants (pathogenic/likely pathogenic) or potentially clinically significant (variants of unknown significance [VUS]). We found 19 clinically significant and potentially clinically significant of HR-DDR gene alterations (germline variants and somatic mutations) in 11 of 18 patients with HRD-positive PDAC (61.1.7%) using WES and WGS (Table S1). Therefore, HR-DDR gene alterations were more frequent in patients with HRD-positive PDAC than in patients with HRD-negative PDAC (Table S6). HR-DDR gene alterations in patients with HRD-positive PDAC are also more likely to be frameshift or nonsense alterations than those identified in patients with HRD-negative PDAC, although missense alterations could still be pathogenic.

To determine whether HRD-positive PDACs are more immunogenic, we compared the tumor mutational burdens (TMBs) and neoantigen loads between HRD-positive and HRD-negative PDACs. We found that HRD-positive PDACs have significantly higher TMBs than HRD-negative tumors (p < 0.0001). Similarly, WES-predicted neoantigen loads that were filtered (p = 0.0002) or not filtered (p = 0.0002) by RNA expression was significantly higher in HRD-positive PDACs than in HRD-negative PDACs (Fig. 1D–F; Table S7). Interestingly, even though HRD is associated with an increased neoantigen load, HRD-positive status (p = 0.1878), higher TMB (p = 0.0906), and higher neoantigen load (p = 0.0041; p = 0.0242), were all associated with a decreasing trend of CD8 + T lymphocytes (Fig. 1G–H; Table S7). We further found that HRD-positive status (p = 0.1321), higher TMB (p = 0.0928), and higher neoantigen load (p = 0.0014; p = 0.0266) are all associated in a trend with a with a decreased T cell diversity (Fig. 4A–D; TableS7). HRD status, TMB, and neoantigen load do not seem to influence clonal distribution and inequality, indicating that there are no particular clonal expansions in HRD-positive, TMB-high, or neoantigen load-high PDACs. Likely due to the small sample size, we did not observe any significant association between the *BRCA1/BRCA2/PALB2* alterations and high TMB or neoantigen load. Consistently, we observed a significantly lower Shannon Diversity Index and a strong trend (p = 0.0910) for unique CDR3 in PDACs with alterations in *BRCA1/BRCA2/PALB2* compared to PDAC without alterations in these genes (p = 0.0291) (Fig. 2E–F; Table S7). Such results may account for the lack of increased effector T cell infiltration in HRD-positive, TMB-high, or neoantigen load-high PDACs.

**Fig. 2:**
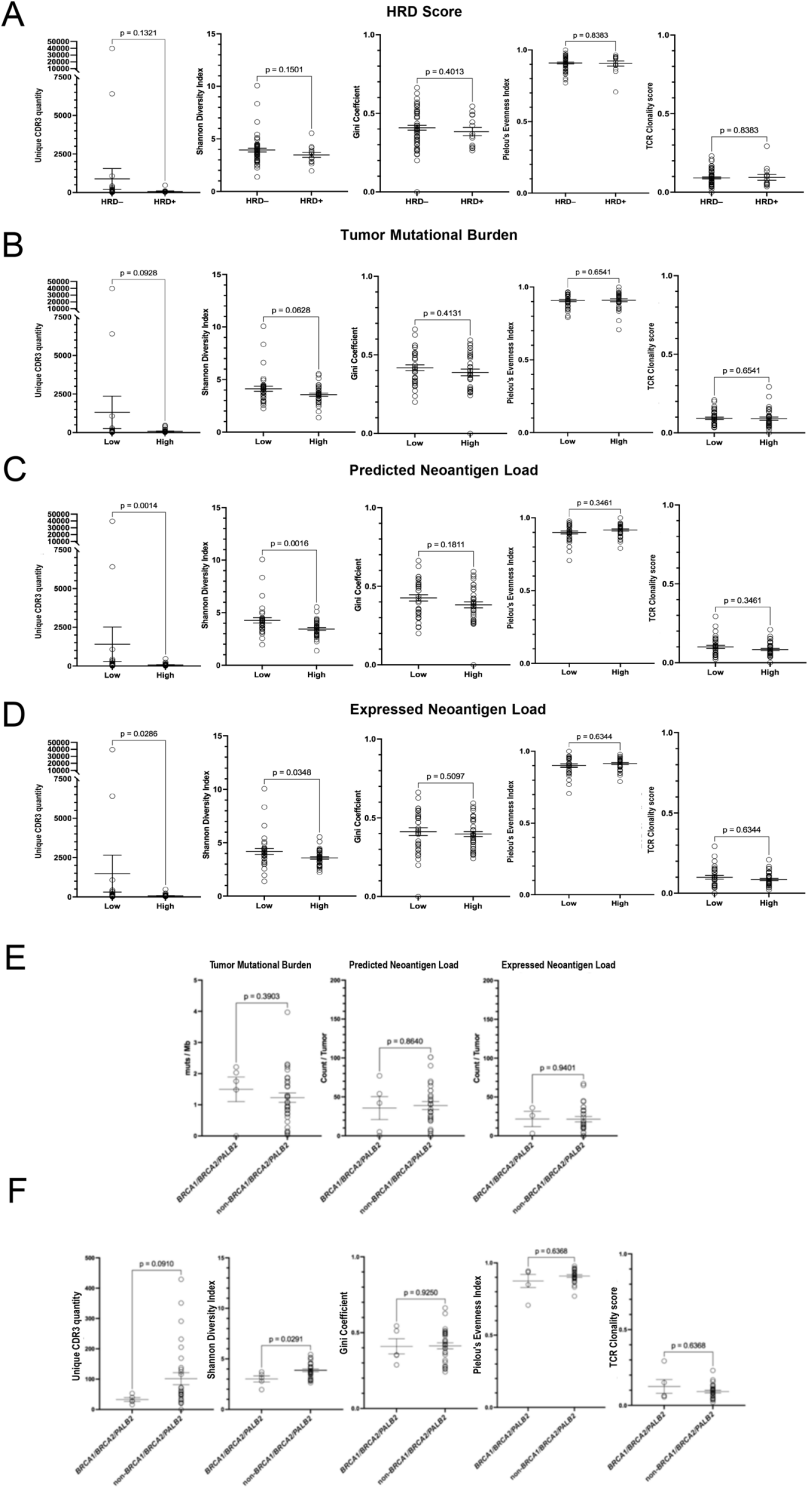
Correlation between TCR repertoires, HRD classification, tumor mutation burden, and neoantigen load and comparisons of TCR repertoires, tumor mutation burden, and neoantigen load between PDACs with and without *BRCA1, BRCA2, and PALB2* gene alterations. PDACs were categorized into HRD positive vs. HRD negative subgroups **A **or divided into “low” vs. “high” subgroups using the median values of tumor mutation burdens **B,** predicted neoantigen load** C,** and expressed neoantigen load **D,** respectively, as classification cutoff. Unique CDR3 quantity, Shannon Diversity Index, Gini Coefficient, Pielou’s Evenness Index, and TCR Clonality score were compared between subgroups, respectively, by t test. Data shown as mean ± SEM. The diversity of the TCR repertoire is predominantly determined by the complementarity-determining region 3 (CDR3), Therefore, the unique number of CDR3 clonotypes per number of TCR reads was used to estimate the clonal diversity. he diversity of the TCR repertoire is predominantly concentrated in the complementarity-determining region 3 (CDR3),The diversity of the TCR repertoire in each sample was also calculated by the Shannon Diversity Index, which took account both the sample richness and the degree of the unevenness of the CDR3 amino acid sequences. The higher the index, ranging from 0 to 1, the more diverse is the CDR3 clones' distribution. The Gini Coefficient Index ranges from 0, which represents maximal diversity of TCR repertoire, to 1, which represents the maximal inequality. The Pielou’s Evenness Index showed whether T cell clones were evenly distributed and ranging from 0 to 1, where 0 represents the least variation in the abundance of clones and 1 represents the greatest variation in the clonal abundance. The complement of clonal evenness, calculated by 1 minus the Pielou’s index, is used to get the TCR Clonality score, ranging from 0, which represents a maximally diverse T cell population, to 1, which represents the most even frequencies in a repertoire driven by clonal dominance, suggesting that there is a clonal expansion. Note that the association between tumor mutation burden (TMB) and number of unique CDR3 was in a strong trend; and that between neoantigen load and number of unique CDR3 was statistically significant. Shannon Diversity Index values showed similar associations. These results suggest that an increased neoantigen load is associated with a decreased T cell diversity. Note that HRD status, TMB, and neoantigen load do not seem to influence clonal distribution and inequality, indicating that there are no particular clonal expansions in HRD-positive, TMB-high, or neoantigen load-high PDACs. Such results may account for the lack of increased effector T cell infiltration in HRD-positive, TMB-high, or neoantigen load-high PDACs. Correlation between HRD status and patient outcomes will be investigated in the future studies. **E.** Tumor mutational burden and neoantigen load were compared between PDACs with *BRCA1, BRCA2, and PALB2* alterations (n = 5) and PDACs without *BRCA1, BRCA2, and PALB2* alterations (n = 30) by t test. Note that the sample size is underpowered. **F.** Unique CDR3 quantity, Shannon Diversity Index, Gini Coefficient, Pielou’s Evenness Index, and TCR Clonality scores were compared between PDACs with and without *BRCA2, and PALB2* alterations, by t test. Data shown as mean ± SEM

Although the increase in TMB we identified in HRD-positive PDACs was small, an increase in neoantigen load suggested that HRD-positive PDAC is potentially immunogenic. This hypothesis would be supported by two recent reports, including one retrospective study of 11 patients with *BRCA1/2*-mutated PDAC who modestly responded to the combination of ipilimumab and nivolumab [11] and the second one showing a small survival benefit when PDAC patients regardless of HRD status were treated with a combination maintenance therapy with a PARP inhibitor niraparib and ipilimumab followed by niraparib and nivolumab [12]. In either study, an HRD analysis was not performed. In our study, HRD-positive PDACs were not found to have an increased effector T cell infiltration or its clonal diversity and expansion. This observation may explain why HRD-positive PDACs do not respond effectively to immune checkpoint inhibitors. Future studies should also be aimed at understanding the immunosuppressive mechanism that limits T cell expansion and the development of an immunotherapy strategy to overcome this resistance mechanism.

## Supplementary Information

Supplementary material 1

Supplementary material 2

Supplementary material 3

Supplementary material 4

## Data Availability

Genomic and transcriptomic data can be accessed at dbGaP with accession number phs003600.

## Abbreviations

PDAC: Pancreatic ductal adenocarcinoma
HRD: Homologous recombination deficiency
DDR: DNA double-strand damage response
ICIs: Immune checkpoint inhibitors
TMB: Tumor mutation burden
TCR: T cell receptor
SNV: Single nucleotide variants
CNV: Copy number variation
SV: Structural variation
LN: Lymph nodes
PLN: Positive lymph nodes
TLN: Total lymph nodes

## References

[1] Pishvaian MJ, Blais EM, Brody JR, Rahib L, Lyons E, De Arbeloa P, et al. Outcomes in patients with pancreatic adenocarcinoma with genetic mutations in DNA damage response pathways: results from the know your tumor program. JCO Precis Oncol. 2019;3:1–10. 35100730 10.1200/PO.19.00115

[2] Li K, Tandurella JA, Gai J, Zhu Q, Lim SJ, Thomas DL 2nd, et al. Multi-omic analyses of changes in the tumor microenvironment of pancreatic adenocarcinoma following neoadjuvant treatment with anti-PD-1 therapy. Cancer Cell. 2022;40(11):1374-91.e7. 36306792 10.1016/j.ccell.2022.10.001 PMC9669212

[3] Wang J, Gai J, Zhang T, Niu N, Qi H, Thomas DL, et al. Neoadjuvant radioimmunotherapy in pancreatic cancer enhances effector T cell infiltration and shortens their distances to tumor cells. Sci Adv. 2024;10(6):1827. 10.1126/sciadv.adk1827 PMC10849596 38324679

[4] Davies H, Glodzik D, Morganella S, Yates LR, Staaf J, Zou X, et al. HRDetect is a predictor of BRCA1 and BRCA2 deficiency based on mutational signatures. Nat Med. 2017;23(4):517–25. 28288110 10.1038/nm.4292 PMC5833945

[5] Nguyen L, Martens JWM, Van Hoeck A, Cuppen E. Pan-cancer landscape of homologous recombination deficiency. Nat Commun. 2020;11(1):5584. 33149131 10.1038/s41467-020-19406-4 PMC7643118

[6] Alexandrov LB, Nik-Zainal S, Wedge DC, Aparicio SA, Behjati S, Biankin AV, et al. Signatures of mutational processes in human cancer. Nature. 2013;500(7463):415–21. 23945592 10.1038/nature12477 PMC3776390

[7] Gulhan DC, Lee JJ-K, Melloni GEM, Cortés-Ciriano I, Park PJ. Detecting the mutational signature of homologous recombination deficiency in clinical samples. Nature Genet. 2019;51(5):912–9. 30988514 10.1038/s41588-019-0390-2

[8] Polak P, Kim J, Braunstein LZ, Karlic R, Haradhavala NJ, Tiao G, et al. A mutational signature reveals alterations underlying deficient homologous recombination repair in breast cancer. Nat Genet. 2017;49(10):1476–86. 28825726 10.1038/ng.3934 PMC7376751

[9] Nik-Zainal S, Davies H, Staaf J, Ramakrishna M, Glodzik D, Zou X, et al. Landscape of somatic mutations in 560 breast cancer whole-genome sequences. Nature. 2016;534(7605):47–54. 27135926 10.1038/nature17676 PMC4910866

[10] Waddell N, Pajic M, Patch AM, Chang DK, Kassahn KS, Bailey P, et al. Whole genomes redefine the mutational landscape of pancreatic cancer. Nature. 2015;518(7540):495–501. 25719666 10.1038/nature14169 PMC4523082

[11] Terrero G, Datta J, Dennison J, Sussman DA, Lohse I, Merchant NB, et al. Ipilimumab/Nivolumab therapy in patients with metastatic pancreatic or biliary cancer with homologous recombination deficiency pathogenic germline variants. JAMA Oncol. 2022;8(6):1–3. 35446342 10.1001/jamaoncol.2022.0611 PMC9026238

[12] Reiss KA, Mick R, Teitelbaum U, O’Hara M, Schneider C, Massa R, et al. Niraparib plus nivolumab or niraparib plus ipilimumab in patients with platinum-sensitive advanced pancreatic cancer: a randomised, phase 1b/2 trial. Lancet Oncol. 2022;23(8):1009–20. 35810751 10.1016/S1470-2045(22)00369-2 PMC9339497

