# Supplementary material for: Homologous recombination-DNA damage response defects increase TMB and neoantigen load, but not effector T cell density and clonal diversity in pancreatic cancer
Source: Exp Hematol Oncol. 2025 Jun 18;14:86. doi: 10.1186/s40164-025-00673-0 (PMC12178057; doi:10.1186/s40164-025-00673-0)
Supplement: Supplementary file 1 — Supplementary material 1 [file 40164_2025_673_MOESM1_ESM.docx]

# Supplemental Materials

**(Supplemental File 1 uploaded)**

# Methods

## Patient specimens

Specimens from a total of 89 patients with pancreatic cancer treated at the Johns Hopkins Hospital, comprised of 41 surgically resectable and 48 locally advanced pancreatic cancer (LAPC) patients, were included for study (**Supplemental Table S1**). Median age of the patients at was 64 years (range 42-85 years). These specimens were archived at the Johns Hopkins Pancreatic Cancer Precision Medicine Center of Excellence Program from two clinical studies (NCT02451982; NCT02648282) according to the Johns Hopkins Medical Institution Institutional Review Board approved registry protocol. Written informed consent to allow the usage of archived specimens for other studies, including this one, was obtained from all the patients through two original clinical studies. Specimens were either endoscopic ultrasound-guided fine needle core biopsies or surgically resected tumor samples (Supplemental Table S3). The demographics of the human cohort were described in previous studies by Li et al. (1) and Wang et al. (2). Thus, this study used tumor specimens archived from two clinical trials of immunotherapy. This approach allowed the inclusion of the tumor specimens collected prospectively, consecutively, and in a uniformed way. As it is possible that patient tumors are affected by study treatments and responses to the study treatments in the clinical trials, the specimens used in this study were all archived prior to any study treatment. Note that, as another potential limitation, specimens used in this study were from archived surgically resectable PDACs and locally advanced PDACs. Nevertheless, an effect of stage of the disease on the results obtained in this study could not be excluded and would need to be discerned in the future studies with a larger sample size.

## DNA extraction, library preparation and sequencing

Genomic DNA and RNA were extracted from freshly frozen biopsy specimens using the AllPrep DNA/RNA Kit (Qiagen) according to the manufacturer's instructions. Tumor biopsy specimens were confirmed at the procedure by cytopathology for the presence of malignant cells. To construct whole-exome capture libraries, genomic DNA was randomly fragmented into 200~250 bp fragments and the fragments were purified and ligated by specific adaptors according to instructions of MGIEasy Universal DNA Library Prep Set, then captured with the MGIEasy Exome Capture V4 Probe Set (~59 Mb; MGI, Shenzhen, China). The WGS libraries were constructed according to instructions of MGIEasy Universal DNA Library Prep Set. All constructed libraries were sequenced on a DIPSEQ platform (BGI). We performed both whole exome sequencing (WES) and whole genome sequencing (WGS) if there was sufficient DNA. Normal control DNA was extracted from peripheral blood mononuclear cells. WES achieved an average coverage of 353X (rang 90 – 592X) in normal samples and 785X (range 83 -1251X) in tumor samples. For WGS datasets, the normal samples achieved an average coverage of 62X (range 23 – 104X), and the tumor samples achieved an average coverage of 64X (range 32 -112X) (Supplemental Table S3).

## Sequencing data analysis

The raw sequencing data were processed using SOAPnuke(RRID:SCR_015025) v2.0.7 (3) to filter low-quality reads and adaptor sequences. The processed reads were then aligned to the UCSC human reference genome (hg19) using Sentieon (4) pipeline with the Sentieon driver. The Sentieon DNA Software package is a speed-up software that rebuilt the phasing algorithms following GATK(RRID:SCR_001876) best practices: read alignment, mark duplication reads, indel realignment, base quality score recalibration (BQSR), and variant calling.

## Calling DNA germline variants

As patients with PDAC often have pathogenic germline variants in homologous repair (HR) and DNA double-strand damage response (DDR) genes, non-somatic variants including Single Nucleotide Polymorphisms (SNPs) and short insertion and deletions (indels) were identified using Sentieon’s Haplotyper algorithm using WES data. The raw SNPs were then filtered by GATK Variant Quality Score Recalibration (VQSR). Raw indels were filtered using the GATK VariantFiltration module with the parameter ‘QD < 2.0 || FS > 200.0 || ReadPosRankSum < -20.0 || SOR > 10.0 || InbreedingCoeff < -0.8.

## *Calling DNA somatic mutations, copy number alterations, and structural variants*

First, Single nucleotide variants (SNVs) and indels were detected using WES and WGS data. We used six somatic mutation callers: Lancet v1.0.7(5), Strelka2 v2.9.2(6), Muse (RRID:SCR_014418) v1.0(7), Mutect2 (GATK4.0.6)(8), Somticsniper v1.0.5.0 (9) and Svaba v0.2.1 (10). The variant calls were refined by intersecting the calls from Mutect2, Strelka2, Lancet, Muse, and Somticsniper for SNVs, and Strelka2, Lancet, Mutect2, and Svaba v0.2.1 for indels. High confident variants were identified by at least two variant callers. Only high-confident calls considered further.

The following criteria, if met, were used to eliminate possible non-somatic artifacts: 1) For variants deposited in the dbSNP(RRID:SCR_002338) database, less than 19 supporting reads in the paired-normal sample or not present in the COSMIC2 database; 2) for variants that were not deposited in dbSNP, less than eight supporting reads in the normal sample; 3) more than 0.1% minor allele frequency (MAF) in the 1000 Genomes Project; 4) more than 0.1% MAF in the ExAc (RRID:SCR_004068) and an annotation in ClinVar that is not “Pathogenic”.

FACETS v0.5.11 (11), an allele-specific copy number algorithm, was used to identify copy number variation (CNV) with default parameters for both WES and WGS data. CNA burden was defined as the percentage of the cancer genome showing copy number changes. Structural variations (SVs) were called from WGS data using Manta(RRID:SCR_022997) v1.5.0 (12) with default parameters. Moreover, a simple reciprocal inversion format was transformed to single inverted sequence junctions by a supplement script provided with Manta (**Supplemental Table S4**).

*Determining the clinical significance of genomic variants*

All SNVs and indels, germline or somatic, of HR-DDR genes were categorized into four groups as described previously (13): (1) Clinically significant variant; (2) VUS potentially clinically significant_a; (3) VUS potentially clinically significant_b; and (4) VUS not clinically significant. First, clinically significant variants were defined as SNV/indel variants with annotations of pathogenic or likely pathogenic in ClinVar27 (GRCh37, database date December 10, 2020). Second, VUS potentially clinically significant_a variants were defined as truncated variants (nonsense, frameshift, or splice intervening sequence ± 1 or 2). Third, VUS potentially clinically significant_b were missense variants that were predicted with any of the three algorithms including MetaSVM (14), MetalLR (14), and FATHMM-MKL (15) to have an outcome of deleterious. Indels with annotations of VUS or had no entry in ClinVar were predicted with SIFT(16) and were categorized as VUS potentially clinically significant_b if the outcomes of SIFT were deleterious. Fourth, VUS not clinically significant were defined as SNV/indel variants annotated as benign or likely benign in ClinVar.

## *Transcriptome sequencing*

RNA libraries were constructed using the MGIEasy rRNA Depletion Kit (MGI, Cat. No. 1000005953) (n = 72). Paired-end sequencing was performed on DIPSEQ platform (BGI). Reads were aligned to the human reference genome hg19 using HISAT (v2.1.1)(17).

## Homologous recombination deficiency score

As BRCA1, BRCA2, and PALB2 are critical components of the homologous recombination (HR) machinery, patients with germline *BRCA1/2*-or *PALB2*-mutated PDAC are categorized as having tumors with homologous recombination deficiency (HRD) (HRD-positive). However, the HR machinery is formed by a number of DNA double-strand damage repair genes, not limited to BRCA1/2 and PALB2(18, 19), and the contribution of individual genes to HRD is unclear. Therefore, to effectively target HRD-positive PDACs, a comprehensive evaluation of HR genes is necessary. Several methods have been developed to assess the HRD status in tumors using genomic features(20). Currently, the only US CAP/CLIA-certified commercial test (myChoiceR CDx assay, Myriad Genetics) is based on the detection of the deleterious mutations in *BRCA1/2* in combination with the Genomic Instability Score (GIS), calculated as the sum of loss of heterozygosity (LOH), large scale state transitions (LST) and telomeric allelic imbalances (TAI) (21) determined using a custom panel of hybridization capture probes targeting single-nucleotide polymorphisms (SNPs) across the genome. There are also several publicly available algorithms to predict HRD status using whole genome sequencing (WGS) or whole exome sequencing (WES). First, scarHRD(22) incorporates the same parameters as the GIS, including LOH, LST, and TAI based on WGS or WES data, which are designated as Genomic Scars. Second, HRDetect(23) combines 6 genomic features based on WGS data, including, 1) deletions with microhomology, 2) two Base Substitution Signatures including Signature 3(24) (25) (enriched in C>G substitutions) and Signature 8(25) (enriched in C>A substitutions) which both lack highly distinctive single-base substitution features among 96 types of base substitutions and are enriched in *BRCA1* and *BRCA2*-null tumors, 3) the Rearrangement Signature 3 which is characterized by short (<10kb) tandem duplications, 4) the Rearrangement Signature 5 which is characterized as non-clustered, 5) <100 kb deletions(26), and 6) a HRD index that is the sum of LOH, TAI, and LST scores. Third, SigMA(27) is based on deleterious mutations in *BRCA1/2* in combination with base substitution Signature 3 using WGS, WES, or targeted gene panel sequence data. Fourth, CHORD is a random forest HRD Classifier based on SNV, indel and structural variation (SV)(28) using WGS data. Finally, one study showed that the HRD subtype exhibited a large number of SV events in PDACs(29).

Myriad myChoice® CDx is highly specific for BRCA1/2-associated HRD and limited to the GIS calculation based on a predefined panel of SNPs. Although publicly available HRD scoring systems can be used to predict HRDs beyond BRCA1/2 mutations, different scoring systems incorporate different types of genomic scars. Although HRDetect combines a more comprehensive list of genomic scars, it is solely based on the results of WGS; thus, it is unable to classify tumors with only WES data available. Therefore, in this study, we developed an HRD classification system below that can utilize all four public-accessible algorithms in combination with SV data based on WGS and/or WES. Where both WGS and WES are available, our method has an enhanced accuracy of HRD prediction.

The scarHRD (22) R package and SigMA (27) were used to calculate the homologous recombination deficiency (HRD) score. For scarHRD, HRD score was calculated according to the sum of the loss of heterozygosity (LOH), telomeric allelic imbalance (TAI), and large-scale state transitions (LSTs). For SigMA, HRD score was calculated according to the mutational signature category. scarHRD and SigMA scores were estimated using WES and WGS data, respectively.

We also used WGS data to estimate HRD scores using HRDetect (23), CHORD (28) and SV numbers(29). The final WES HRD score in this study was defined as the sum of scarHRD and SigMA HRD scores based on WES data, while the final WGS HRD score in this study was defined as the sum of scarHRD, SigMA, HRDetect, CHORD and SV HRD scores based on WGS data.

A tumor is considered to be HRD (HRD-positive) if the WES HRD score >2 (as the HRD category 1; c1), if the WGS HRD score >2 (as the HRD category 2; c2): or if the WES HRD score >0 and the WGS HRD score >1 (as the HRD category 3; c3) (Supplemental Figure1). We use those published tools and thresholds defined by each tool. Either WES or WGS score >2 would mean that HRD can be positively detected by at least one tool using q strict threshold and a second tool using a loose threshold or HRD can be positively detected by at least three tools using a loose threshold. Either WES or WGS score >2 would meet the criteria for HRD. For those samples with both WES and WGS, WES score >0 and WGS score >1 would mean that HRD is positively detected by at least one WES tool using a loose threshold and HRD is positively detected by at least two WGS tools using a loose threshold.

## Calculation of tumor mutation burden and neoantigen load

The tumor mutation burden (TMB) was calculated by the total number of somatic non-synonymous SNV and INDEL mutations per megabase in the coding region using the WES data. HLA-allele typing was predicted by OptiType v1.3.1 (30) using the WES results. Non-synonymous SNVs and INDEL were translated into 9~11 amino acids with a sliding window method as the candidate mutated peptides. NetMHC v4.0 (31), NetMHCpan v4.1b (32), MHCflurry v 2.0.1 (33) and MixMHCpred v2.1 (34) were used to predict the HLA binding peptides. The final neoantigens were retained if at least two of the following four criteria were met: 1) NetMHC with binding affinity <=500nM, 2) NetMHCpan with binding affinity <=500nM, 3) MHCflurry with binding affinity <=500nM and presentation score =0.5, 4) MixMHCpred with Score_bestAllele >=0 and best allele consist with HLA type. Neoantigen load was estimated as the number of peptides that bind to the HLA-A allele. If RNA sequencing data were available, neoantigens were further filtered based on gene expression level (FPKM >= 1). Whole transcriptome RNA sequencing (RNA-seq) was available for 72 of 89 patient specimens. Two samples with low-purity sequencing results showed low TMB. As low TMB can be an artifact due to the low purity, these two samples were excluded from further analysis (**Supplemental Table S7**).

## Diversity of T cell receptor (TCR) repertoire

In T-cell repertoires, diversity relates to the level of uncertainty that a TCR sequence would be sorted from a repertoire and would belong to a unique T-cell clone. Thus, as described by Chiffelle et al (35), the diversity measurement considers the clonal composition, equivalent to the number of unique TCR sequences designated richness and the distribution spectrum of these sequences designated evenness. Here, MiXCR (36) was used to process the raw sequences of RNA for quantitating the clonotypes of patients. The diversity of the TCR repertoire is predominantly determined by the complementarity-determining region 3 (CDR3), Therefore, the unique number of CDR3 clonotypes per number of TCR reads was used to estimate the clonal diversity. The diversity of the TCR repertoire in each sample was also calculated by the Shannon Diversity Index (35), which took account both the sample richness and the degree of the unevenness of the CDR3 amino acid sequences. The higher the index, ranging from 0 to 1, the more diverse is the CDR3 clones' distribution. The Gini Coefficient Index (35) ranges from 0, which represents maximal diversity of TCR repertoire, to 1, which represents the maximal inequality. The Pielou’s Evenness Index (35) showed whether T cell clones were evenly distributed and ranging from 0 to 1, where 0 represents the least variation in the abundance of clones and 1 represents the greatest variation in the clonal abundance. The complement of clonal evenness, calculated by 1 minus the Pielou’s index, is used to get the TCR Clonality score, ranging from 0, which represents a maximally diverse T cell population, to 1, which represents the most even frequencies in a repertoire driven by clonal dominance, suggesting that there is a clonal expansion.

## Sequential multiplex IHC

The sequential multiplex IHC (mIHC) was performed on biopsy specimens before the patients received any experimental therapy. The method to analyze the percentage of immune cell infiltration and markers to identify immune cells was described in the study by Li et al. (1) and by Wang et al.(2) The published quantitative immune cell density results, reported as the percent among all cells in the tumor areas defined by pathologist (**Supplemental Table S7**), were used without any modification for the analysis in this study. 59 out of 89 patients have mIHC quantitative results available for this study.

## Statistical analysis

Specimens were grouped into two groups, one having “high” and the other having “low” density of immune cell subtypes as indicated, TMB, and neoantigen load, respectively, by using the respective median value as the cutoff, or according to the HRD status as above described. The Student's t tests were performed to compare ordinal datas between the two independent groups. A p value < 0.05 was considered statistically significant. Statistical analyses and graphs were generated using GraphPad Prism(RRID:SCR_002798) v.9.3.1.

**References in Supplemental Materials**

1. Li K, Tandurella JA, Gai J, Zhu Q, Lim SJ, Thomas DL, 2nd, et al. Multi-omic analyses of changes in the tumor microenvironment of pancreatic adenocarcinoma following neoadjuvant treatment with anti-PD-1 therapy. Cancer Cell. 2022;40(11):1374-91.e7.

2. Wang J, Gai J, Zhang T, Niu N, Qi H, Thomas DL, 2nd, et al. Neoadjuvant radioimmunotherapy in pancreatic cancer enhances effector T cell infiltration and shortens their distances to tumor cells. Sci Adv. 2024;10(6):eadk1827.

3. Chen Y, Chen Y, Shi C, Huang Z, Zhang Y, Li S, et al. SOAPnuke: a MapReduce acceleration-supported software for integrated quality control and preprocessing of high-throughput sequencing data. Gigascience. 2018;7(1):1-6.

4. Forbes SA, Beare D, Boutselakis H, Bamford S, Bindal N, Tate J, et al. COSMIC: somatic cancer genetics at high-resolution. Nucleic Acids Res. 2017;45(D1):D777-d83.

5. Narzisi G, Corvelo A, Arora K, Bergmann EA, Shah M, Musunuri R, et al. Genome-wide somatic variant calling using localized colored de Bruijn graphs. Communications biology. 2018;1:20.

6. Kim S, Scheffler K, Halpern AL, Bekritsky MA, Noh E, Källberg M, et al. Strelka2: fast and accurate calling of germline and somatic variants. Nature methods. 2018;15(8):591-4.

7. Fan Y, Xi L, Hughes DST, Zhang J, Zhang J, Futreal PA, et al. MuSE: accounting for tumor heterogeneity using a sample-specific error model improves sensitivity and specificity in mutation calling from sequencing data. Genome Biology. 2016;17(1):178.

8. Cibulskis K, Lawrence MS, Carter SL, Sivachenko A, Jaffe D, Sougnez C, et al. Sensitive detection of somatic point mutations in impure and heterogeneous cancer samples. Nature Biotechnology. 2013;31(3):213-9.

9. Larson DE, Harris CC, Chen K, Koboldt DC, Abbott TE, Dooling DJ, et al. SomaticSniper: identification of somatic point mutations in whole genome sequencing data. Bioinformatics. 2012;28(3):311-7.

10. Wala JA, Bandopadhayay P, Greenwald NF, O'Rourke R, Sharpe T, Stewart C, et al. SvABA: genome-wide detection of structural variants and indels by local assembly. Genome Res. 2018;28(4):581-91.

11. Shen R, Seshan VE. FACETS: allele-specific copy number and clonal heterogeneity analysis tool for high-throughput DNA sequencing. Nucleic Acids Res. 2016;44(16):e131.

12. Chen X, Schulz-Trieglaff O, Shaw R, Barnes B, Schlesinger F, Källberg M, et al. Manta: rapid detection of structural variants and indels for germline and cancer sequencing applications. Bioinformatics. 2016;32(8):1220-2.

13. Xie F, Ding D, Lin C, Cunningham D, Wright M, Javed AA, et al. RAD51B Harbors Germline Mutations Associated With Pancreatic Ductal Adenocarcinoma. JCO precision oncology. 2022;6:e2100404.

14. Dong C, Wei P, Jian X, Gibbs R, Boerwinkle E, Wang K, et al. Comparison and integration of deleteriousness prediction methods for nonsynonymous SNVs in whole exome sequencing studies. Hum Mol Genet. 2015;24(8):2125-37.

15. Shihab HA, Rogers MF, Gough J, Mort M, Cooper DN, Day IN, et al. An integrative approach to predicting the functional effects of non-coding and coding sequence variation. Bioinformatics. 2015;31(10):1536-43.

16. Hu J, Ng PC. SIFT Indel: predictions for the functional effects of amino acid insertions/deletions in proteins. PLoS One. 2013;8(10):e77940.

17. Rakocevic G, Semenyuk V, Lee W-P, Spencer J, Browning J, Johnson IJ, et al. Fast and accurate genomic analyses using genome graphs. Nature Genetics. 2019;51(2):354-62.

18. Doig KD, Fellowes AP, Fox SB. Homologous Recombination Repair Deficiency: An Overview for Pathologists. Mod Pathol. 2023;36(3):100049.

19. Liu J, Mroczek M, Mach A, Stepien M, Aplas A, Pronobis-Szczylik B, et al. Genetics, Genomics and Emerging Molecular Therapies of Pancreatic Cancer. Cancers (Basel). 2023;15(3).

20. Doig KD, Fellowes AP, Fox SB. Homologous Recombination Repair Deficiency: An Overview for Pathologists. Modern Pathology. 2023;36(3).

21. Timms KM, Abkevich V, Hughes E, Neff C, Reid J, Morris B, et al. Association of BRCA1/2 defects with genomic scores predictive of DNA damage repair deficiency among breast cancer subtypes. Breast cancer research : BCR. 2014;16(6):475.

22. Sztupinszki Z, Diossy M, Krzystanek M, Reiniger L, Csabai I, Favero F, et al. Migrating the SNP array-based homologous recombination deficiency measures to next generation sequencing data of breast cancer. NPJ Breast Cancer. 2018;4:16.

23. Davies H, Glodzik D, Morganella S, Yates LR, Staaf J, Zou X, et al. HRDetect is a predictor of BRCA1 and BRCA2 deficiency based on mutational signatures. Nature Medicine. 2017;23(4):517-25.

24. Alexandrov LB, Nik-Zainal S, Wedge DC, Aparicio SA, Behjati S, Biankin AV, et al. Signatures of mutational processes in human cancer. Nature. 2013;500(7463):415-21.

25. Polak P, Kim J, Braunstein LZ, Karlic R, Haradhavala NJ, Tiao G, et al. A mutational signature reveals alterations underlying deficient homologous recombination repair in breast cancer. Nature Genetics. 2017;49(10):1476-86.

26. Nik-Zainal S, Davies H, Staaf J, Ramakrishna M, Glodzik D, Zou X, et al. Landscape of somatic mutations in 560 breast cancer whole-genome sequences. Nature. 2016;534(7605):47-54.

27. Gulhan DC, Lee JJ-K, Melloni GEM, Cortés-Ciriano I, Park PJ. Detecting the mutational signature of homologous recombination deficiency in clinical samples. Nature Genetics. 2019;51(5):912-9.

28. Nguyen L, J WMM, Van Hoeck A, Cuppen E. Pan-cancer landscape of homologous recombination deficiency. Nat Commun. 2020;11(1):5584.

29. Waddell N, Pajic M, Patch AM, Chang DK, Kassahn KS, Bailey P, et al. Whole genomes redefine the mutational landscape of pancreatic cancer. Nature. 2015;518(7540):495-501.

30. Szolek A, Schubert B, Mohr C, Sturm M, Feldhahn M, Kohlbacher O. OptiType: precision HLA typing from next-generation sequencing data. Bioinformatics. 2014;30(23):3310-6.

31. Andreatta M, Nielsen M. Gapped sequence alignment using artificial neural networks: application to the MHC class I system. Bioinformatics. 2016;32(4):511-7.

32. Jurtz V, Paul S, Andreatta M, Marcatili P, Peters B, Nielsen M. NetMHCpan-4.0: Improved Peptide-MHC Class I Interaction Predictions Integrating Eluted Ligand and Peptide Binding Affinity Data. J Immunol. 2017;199(9):3360-8.

33. O'Donnell TJ, Rubinsteyn A, Laserson U. MHCflurry 2.0: Improved Pan-Allele Prediction of MHC Class I-Presented Peptides by Incorporating Antigen Processing. Cell Syst. 2020;11(1):42-8.e7.

34. Gfeller D, Guillaume P, Michaux J, Pak H-S, Daniel RT, Racle J, et al. The Length Distribution and Multiple Specificity of Naturally Presented HLA-I Ligands. The Journal of Immunology. 2018;201(12):3705-16.

35. Chiffelle J, Genolet R, Perez MA, Coukos G, Zoete V, Harari A. T-cell repertoire analysis and metrics of diversity and clonality. Curr Opin Biotechnol. 2020;65:284-95.

36. Bolotin DA, Poslavsky S, Mitrophanov I, Shugay M, Mamedov IZ, Putintseva EV, et al. MiXCR: software for comprehensive adaptive immunity profiling. Nature methods. 2015;12(5):380-1.

**Supplemental Figures and Tables**

**Supplemental Figure 1. HRD classification based on Homologous Recombination Deficiency Score**

**
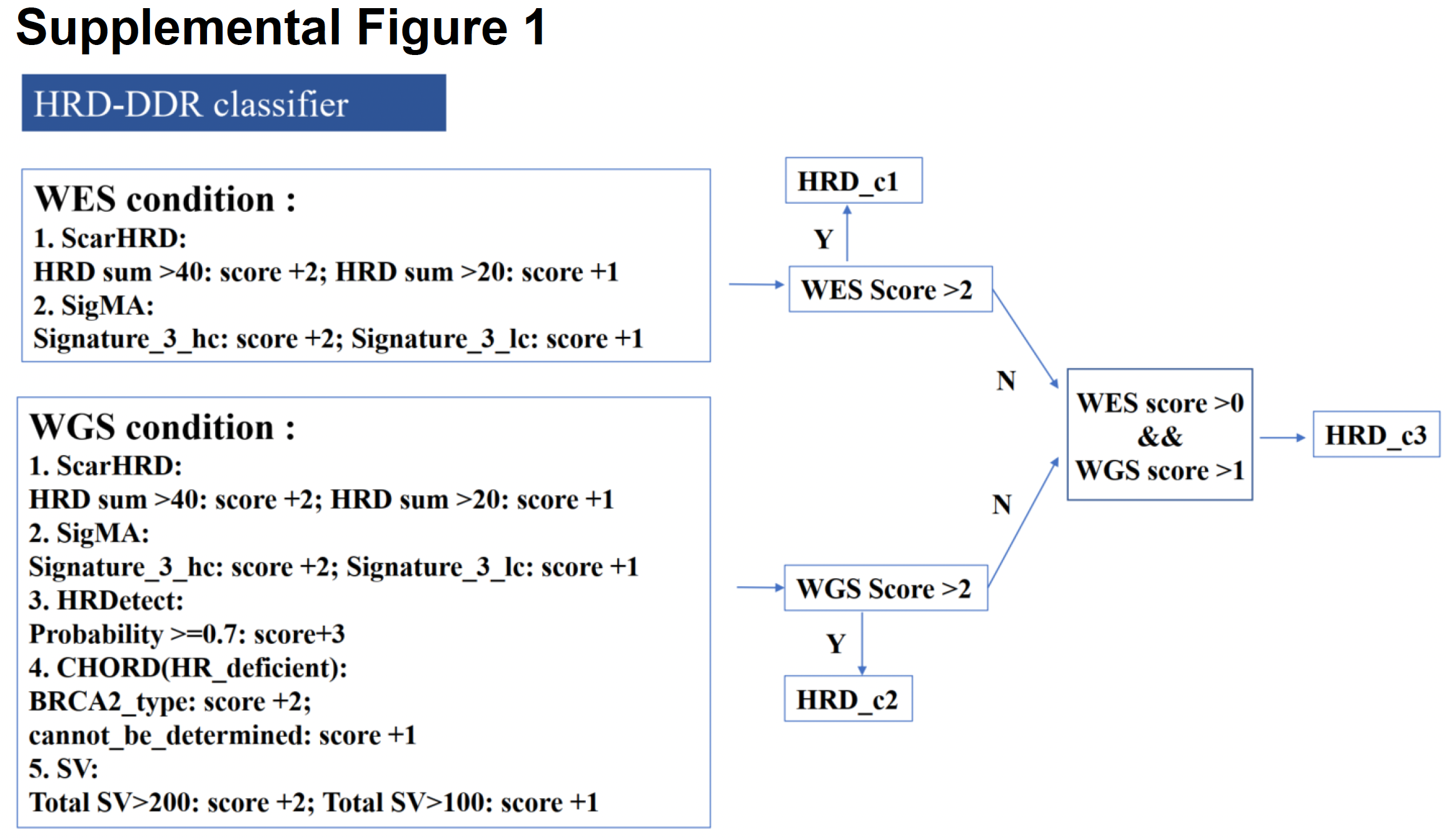
**

Signature_3_hc indicates that the sample passes the strict threshold. Signature_3_lc indicates that the sample passes the looser threshold but not the strict threshold.

## Supplemental Table 1. Demographic and clinicopathologic characteristics

|  | **Total *N =* 89 *** | |  |
| --- | --- | --- | --- |
| **Variables** | **HRD-negative PDAC (*n* = 69)** | **HRD-positive PDAC (*n* = 18)** | **Significance** |
| **Age at surgery, years, *n* (%)**  >=65  <65 |  |  |  |
|  | 36 (52) | 10 (56) |  |
|  | 33 (48) | 8 (44) | *p* = 0.798 |
| **Sex, *n* (%)**  Male  Female |  |  |  |
|  | 39 (57) | 10 (56) |  |
|  | 30 (43) | 8 (44) | *p* = 0.941 |
| **Stage, *n* (%)**  Surgically resectable  Locally advanced |  |  |  |
|  | 29 (42) | 10 (56) |  |
|  | 40 (58) | 8 (44) | *p* = 0.304 |
| **Histologic Grade, *n* (%) ****  G1: Well differentiated  G2: Moderately differentiated  G2/G3: Moderately/Poorly differentiated  G3: Poorly differentiated  G4: Undifferentiated |  |  |  |
|  | 2 (3) | 0 (0) |  |
|  | 43 (62) | 11 (61) |  |
|  | 2 (3) | 1 (6) |  |
|  | 17 (25) | 3 (17) |  |
|  | 0 (0) | 0 (0) | *p* = 0.629 |
| **Tumor Size, cm *****  Median (IQR) |  |  |  |
|  | 2.7 (1.70) | 3.2 (1.40) | *p* = 0.741 |
| **Primary Tumor Stage (y, at surgical resection),**  ***n* (% resected patients)**  pT0  pT1  pT2  pT3  pT4 |  |  |  |
|  | 2 (4) | 0 (0) |  |
|  | 10 (22) | 2 (15) |  |
|  | 16 (36) | 4 (31) |  |
|  | 12 (27) | 6 (46) |  |
|  | 5 (11) | 1 (8) | *p* = 0.751 |
| **Margins, *n* (% resected patients) ******  Uninvolved  Involved |  |  |  |
|  | 37 (82) | 13 (100) |  |
|  | 7 (16) | 0 (0) | *p* = 0.225 |
| **LN, *n* (% resected patients)**  Negative  Positive |  |  |  |
|  | 21 (47) | 7 (54) |  |
|  | 24 (53) | 6 (46) | *p* = 0.765 |
| **PLN**  Median (IQR) |  |  |  |
|  | 0 (2.25) | 0 (1.00) | *p* = 0.231 |
| **TLN**  Median (IQR) |  |  |  |
|  | 20 (11.00) | 23 (3.50) | *p* = 0.129 |

Abbreviations: LN, lymph nodes; PLN, positive lymph nodes; TLN, total lymph nodes. * Two cases were excluded due to low depth of sequencing. ** Histologic grade is unknown for eight patients. Margin status is unknown for one patient. *** No residual viable adenocarcinoma at surgical resection in two patients.

## Supplemental Table 2. Homologous Recombination, DNA Damage Response, and Repair Gene Variants in Pancreatic Ductal Adenocarcinoma

| **HRD**  **classification** | **Patient ID** | **Gene*** | **Genome Change** | **Protein Change** | **Variant Allele Frequency** | **Germline v Somatic** |
| --- | --- | --- | --- | --- | --- | --- |
| HRD_c2&&HRD_c1 | P01 |  |  |  |  |  |
| HRD_c2 | P02 |  |  |  |  |  |
| HRD_c1 | P03 | *BRCA2* | 13:g.32914066_32914069del | p.I1859Kfs*3 | 0.52 | Germline |
| HRD_c1 |  | *BRCA2* | 13:g32937655_32937656insATCTCTT | p.M2775Ifs*9 | 0.04 | Somatic |
| HRD_c2 | P04 | *BRCA2* | 13:g.32920968_32920971del | p.I2315Kfs*12 | 0.52 | Germline |
|  |  | *BRCA2* | 13:g.32931983G>A | p.W2574* | 0.06 | Somatic |
|  |  | *RBBP8* | 18:g.20573157A>G | p.H456R | 0.52 | Germline |
| HRD_c3 | P05 | *RAD51B* | 14:g.68331815_68331819del | p.V139Hfs*2 | 0.55 | Germline |
| HRD_c3 | P06 |  |  |  |  |  |
| HRD_c2 | P07 | *UIMC1* | 5:g.176396076T>C | p.H227R | 0.57 | Germline |
| HRD_c1 | P08 | *ATM* | 11:g.108141988T>C | p.S978P | 0.51 | Germline |
|  |  | *ATM* | 11:g.108188118C>T | p.L2073F | 0.57 | Germline |
| HRD_c3 | P09 |  |  |  |  |  |
| HRD_c2 | P10 | *ATM* | 11:g.108186610G>A | p.G2023R | 0.57 | Germline |
|  |  | *BRCA1* | 17:g.41245239G>T | p.S770* | 0.39 | Germline |
|  |  | *POLD1* | 19:g.50909491G>A | p.R432Q | 0.56 | Germline |
| HRD_c2 | P11 |  |  |  |  |  |
| HRD_c2 | P12 | *ATR* | 3:g.142234329A>C | p.W1471G | 0.46 | Germline |
| HRD_c2 | P13 | *BRCA2* | 13:g.32900683_32900684insG | p.D189Gfs*3 | 0.60 | Germline |
| HRD_c2 | P14 | *MUS81* | 11:g.65632507G>A | p.R431H | 0.49 | Germline |
|  |  | *RBBP8* | 18:g.20602158C>A | p.R841S | 0.55 | Germline |
| HRD_c2 | P15 | *ATM* | 11:g.108216545C>T | p.R2832C | 0.39 | Germline |
|  |  | *RAD51B* | 14:g.68290267A>G | p.S3G | 0.53 | Germline |
| HRD_c2 | P16 |  |  |  |  | Germline |
|  |  |  |  |  |  | Germline |
|  |  | *BACH1* | 21:g.30699137A>G | p.Q331R | 0.47 | Germline |
| HRD_c2 | P17 |  |  |  |  |  |
| HRD_c2 | P18 |  |  |  |  |  |

* exclude VUS not clinically significant and benign variants.

Note that among the 11 patients with HRD-positive PDAC, three patients had three different HR-DDR genes alterations; four patients had two different alterations; and the remaining five patients each had one alteration. Of the 19 HR-DDR gene alterations identified, the majority were missense alterations (N=12, 63.2%), followed by five frameshift alterations (26.3%) and two nonsenses alterations (10.5%). Alterations were identified in 11 HR-DDR genes, with alterations most frequently identified in *BRCA2* (N=3) and *ATM* (N=3),followed by *RAD51B* (N=2). In 23 of 69 (33.33%) patients with HRD-negative PDAC, we identified 28 HR-DDR gene alterations classified as not clinically significant **(Supplemental Table S6).** Among these 23 patients with HRD- negative PDAC, five patients had two different alterations in the HR-DDR genes, while the remaining eighteen patients each had one alteration. Of the 28 gene alterations identified, the majority were missense alterations (N=25, 89.3%), followed by two frameshift alterations (7.1%) and one nonsense alteration (3.6%). Alterations in patients with HRD- negative PDAC were identified in 18 HR-DDR genes, with alterations most frequently identified in *ATM* (N=3; 10.7%) and *RAD54L* (N=3; 10.7%).

**Supplemental Table 3. Summary statistics of whole genome and whole exome sequencing data**

**(Supplemental File 2 uploaded)**

**Supplemental Table 4. Genomic alterations of whole genome and whole exome sequencing data**

**(Supplemental File 3 uploaded)**

## Supplemental Table 5. HRD-DDR classification

| **Patient ID** | **HRD classification** | **WES Score** | **ScarHRD** | **SigMA** | **WGS Score** | **ScarHRD** | **SigMA** | **HRDetect** | **CHORD** | **SV** |
| --- | --- | --- | --- | --- | --- | --- | --- | --- | --- | --- |
| P01 | HRD_c2&&HRD_c1 | 3 | 23 | Signature_3_hc | 10 | 47 | Signature_3_hc | 0.998 | HR_deficient (BRCA2_type) | 165 |
| P02 | HRD_c2 | 1 | 32 |  | 4 | 42 |  | 0.002 |  | 234 |
| P03 | HRD_c1 | 4 | 46 | Signature_3_hc |  |  |  |  |  |  |
| P04 | HRD_c2 | 2 | 1 | Signature_3_hc | 10 | 61 | Signature_3_hc | 0.997 | HR_deficient (BRCA2_type) | 104 |
| P05 | HRD_c3 | 2 | 42 |  | 2 | 39 |  | 0.006 |  | 113 |
| P06 | HRD_c3 | 1 | 29 |  | 2 | 28 |  | 0.000 |  | 152 |
| P07 | HRD_c2 | 1 | 31 |  | 3 | 48 |  | 0.004 |  | 162 |
| P08 | HRD_c1 | 3 | 26 | Signature_3_hc |  |  |  |  |  |  |
| P09 | HRD_c3 | 2 | 41 |  | 2 | 55 |  | 0.000 |  | 41 |
| P10 | HRD_c2 | 1 | 26 |  | 4 | 16 | Signature_3_hc | 0.095 | HR_deficient (BRCA2_type) | 91 |
| P11 | HRD_c2 | 2 | 48 |  | 6 | 45 | Signature_3_hc | 0.018 |  | 225 |
| P12 | HRD_c2 | 2 | 44 |  | 3 | 44 |  | 0.068 |  | 136 |
| P13 | HRD_c2 | 1 | 24 |  | 4 | 24 | Signature_3_hc | 0.002 |  | 148 |
| P14 | HRD_c2 | 2 | 12 | Signature_3_hc | 3 | 23 |  | 0.005 |  | 617 |
| P15 | HRD_c2 | 1 | 28 |  | 3 | 23 |  | 0.001 |  | 206 |
| P16 | HRD_c2 | 0 | 6 |  | 3 | 29 |  | 0.001 |  | 287 |
| P17 | HRD_c2 | 0 | 10 |  | 3 | 10 | Signature_3_lc | 0.000 |  | 450 |
| P18 | HRD_c2 | 2 | 48 |  | 3 | 3 | Signature_3_hc | 0.000 |  | 103 |
| P19 |  | 1 | 4 | Signature_3_lc |  |  |  |  |  |  |
| P20 |  | 1 | 21 |  |  |  |  |  |  |  |
| P21 |  | 1 | 33 |  | 1 | 28 |  | 0.137 |  | 70 |
| P22 |  | 0 | 4 |  | 2 | 28 |  | 0.001 |  | 195 |
| P23 |  | 1 | 38 |  |  |  |  |  |  |  |
| P24 |  | 1 | 29 |  | 0 | 17 |  | 0.001 |  | 21 |
| P25 |  | 0 | 18 |  |  |  |  |  |  |  |
| P26 |  | 2 | 57 |  |  |  |  |  |  |  |
| P27 |  | 0 | 11 |  |  |  |  |  |  |  |
| P28 |  | 1 | 35 |  |  |  |  |  |  |  |
| P29 |  | 1 | 9 | Signature_3_lc | 0 | 1 |  | 0.002 |  | 92 |
| P30 |  | 2 | 54 |  |  |  |  |  |  |  |
| P31 |  | 1 | 38 |  |  |  |  |  |  |  |
| P32 |  | 0 | 0 |  |  |  |  |  |  |  |
| P33 |  | 1 | 23 |  |  |  |  |  |  |  |
| P34 |  | 0 | 11 |  |  |  |  |  |  |  |
| P35 |  | 2 | 57 |  |  |  |  |  |  |  |
| P36 |  | 0 | 5 |  | 1 | 16 |  | 0.004 |  | 127 |
| P37 |  | 0 | 6 |  | 1 | 31 |  | 0.003 |  | 81 |
| P38 |  | 0 | 5 |  | 0 | 1 |  | 0.000 |  | 19 |
| P39 |  | 0 | 2 |  |  |  |  |  |  |  |
| P40 |  | 0 | 6 |  |  |  |  |  |  |  |
| P41 |  | 0 | 20 |  |  |  |  |  |  |  |
| P42 |  | 0 | 11 |  | 0 | 11 |  | 0.000 |  | 24 |
| P43 |  | 1 | 31 |  | 1 | 29 |  | 0.000 |  | 39 |
| P44 |  | 0 | 2 |  | 0 | 0 |  | 0.000 |  | 28 |
| P45 |  | 1 | 32 |  | 1 | 23 |  | 0.003 |  | 48 |
| P46 |  | 1 | 38 |  | 0 | 10 |  | 0.007 |  | 53 |
| P47 |  | 0 | 17 |  | 0 | 15 |  | 0.000 |  | 58 |
| P48 |  | 0 | 6 |  |  |  |  |  |  |  |
| P49 |  | 1 | 26 |  | 0 | 20 |  | 0.001 |  | 29 |
| P50 |  | 0 | 3 |  | 0 | 19 |  | 0.002 |  | 28 |
| P51 |  | 1 | 21 |  |  |  |  |  |  |  |
| P52 |  | 0 | 2 |  | 2 | 16 |  | 0.188 | HR_deficient (BRCA2_type) | 64 |
| P53 |  | 0 | 19 |  |  |  |  |  |  |  |
| P54 |  | 2 | 41 |  | 1 | 21 |  | 0.001 |  | 31 |
| P55 |  | 0 | 2 |  | 0 | 5 |  | 0.000 |  | 23 |
| P56 |  | 0 | 1 |  |  |  |  |  |  |  |
| P57 |  | 0 | 1 |  |  |  |  |  |  |  |
| P58 |  | 1 | 31 |  | 1 | 31 |  | 0.003 |  | 39 |
| P59 |  | 0 | 6 |  |  |  |  |  |  |  |
| P60 |  | 0 | 20 |  | 2 | 21 |  | 0.001 |  | 164 |
| P61 |  | 0 | 2 |  | 0 | 14 |  | 0.000 |  | 12 |
| P62 |  | 2 | 48 |  | 1 | 33 |  | 0.001 |  | 31 |
| P63 |  | 0 | 1 |  |  |  |  |  |  |  |
| P64 |  | 0 | 12 |  | 0 | 0 |  | 0.001 |  | 12 |
| P65 |  | 0 | 17 |  | 0 | 0 |  | 0.000 |  | 14 |
| P66 |  | 0 | 2 |  |  |  |  |  |  |  |
| P67 |  | 0 | 1 |  |  |  |  |  |  |  |
| P68 |  | 0 | 1 |  | 0 | 5 |  | 0.000 |  | 20 |
| P69 |  | 0 | 13 |  |  |  |  |  |  |  |
| P70 |  | 0 | 17 |  | 0 | 0 |  | 0.177 |  | 5 |
| P71 |  | 0 | 2 |  | 1 | 0 |  | 0.013 | HR_deficient (cannot_be_determined) | 19 |
| P72 |  | 0 | 18 |  |  |  |  |  |  |  |
| P73 |  | 0 | 1 |  | 2 | 0 |  | 0.078 | HR_deficient (BRCA2_type) | 52 |
| P74 |  | 0 | 4 |  |  |  |  |  |  |  |
| P75 |  | 0 | 4 |  | 1 | 5 |  | 0.080 |  | 186 |
| P76 |  | 0 | 3 |  |  |  |  |  |  |  |
| P77 |  | 0 | 3 |  | 0 | 6 |  | 0.000 |  | 6 |
| P78 |  | 0 | 1 |  |  |  |  |  |  |  |
| P81 |  | 0 | 8 |  | 0 | 0 |  | 0.000 |  | 2 |
| P82 |  | 0 | 2 |  |  |  |  |  |  |  |
| P83 |  | 0 | 3 |  |  |  |  |  |  |  |
| P84 |  | 0 | 4 |  |  |  |  |  |  |  |
| P85 |  | 0 | 1 |  |  |  |  |  |  |  |
| P86 |  | 0 | 2 |  |  |  |  |  |  |  |
| P87 |  | 0 | 4 |  | 0 | 0 |  | 0.001 |  | 2 |
| P88 |  | 1 | 23 |  | 0 | 0 |  | 0.000 |  | 1 |
| P89 |  | 0 | 1 |  | 0 | 1 |  | 0.002 |  | 1 |

## Supplemental Table 6. HR-DDR gene alterations in HRD-negative Pancreatic Ductal Adenocarcinoma

| **Patient ID** | **Gene** | **Genome Change** | **Protein Change** | **Variant Allele Frequency** | **Germline or Somatic** |
| --- | --- | --- | --- | --- | --- |
| P19 |  |  |  |  |  |
| P20 | *MUS81* | 11:g.65632242G>A | p.A410T | 0.08 | Somatic |
| P20 | *BLM* | 15:g.91341438G>A | p.V1077M | 0.09 | Somatic |
| P21 |  |  |  |  |  |
| P22 |  |  |  |  |  |
| P23 | *XRCC2* | 7:g.152345797C>T | p.R258H | 0.52 | Germline |
| P24 | *ATM* | 11:g.108119666del | p.N358Mfs*32 | 0.32 | Germline |
| P25 | *FANCC* | 9:g.97873790G>C | p.F428L | 0.11 | Germline |
| P26 |  |  |  |  |  |
| P27 |  |  |  |  |  |
| P28 |  |  |  |  |  |
| P29 |  |  |  |  |  |
| P30 | *RAD51B* | 14:g.68352648T>G | p.L172W | 1 | Germline |
| P31 | *XRCC2* | 7:g.152345927G>A | p.R215* | 0.42 | Germline |
| P31 | *FANCM* | 14:g.45623953T>C | p.Y413H | 0.5 | Germline |
| P32 |  |  |  |  |  |
| P33 |  |  |  |  |  |
| P34 |  |  |  |  |  |
| P35 |  |  |  |  |  |
| P36 | *UIMC1* | 5:g.176335682A>G | p.Y564H | 0.49 | Germline |
| P37 |  |  |  |  |  |
| P38 |  |  |  |  |  |
| P39 |  |  |  |  |  |
| P40 |  |  |  |  |  |
| P41 | *RAD54L* | 1:g.46739120T>C | p.L490P | 0.48 | Germline |
| P42 | *MUS81* | 11:g.65632507G>A | p.R431H | 0.10 | Germline |
| P42 | *BRCC3* | X:g.154305469A>G | p.I74V | 1 | Germline |
| P43 |  |  |  |  |  |
| P44 | *RAD54L* | 1:g.46743923G>A | p.R738H | 0.38 | Germline |
| P45 | *RAD51D* | 17:g.33428040C>T | p.E327K | 0.48 | Germline |
| P46 |  |  |  |  |  |
| P47 | *RAD51B* | 14:g.68353913T>G | p.S250A | 0.47 | Germline |
| P48 |  |  |  |  |  |
| P49 |  |  |  |  |  |
| P50 |  |  |  |  |  |
| P51 |  |  |  |  |  |
| P52 | *BACH1* | 21:g.30699557G>C | p.G471A | 0.50 | Germline |
| P53 |  |  |  |  |  |
| P54 |  |  |  |  |  |
| P55 |  |  |  |  |  |
| P56 |  |  |  |  |  |
| P57 | *POLD1* | 19:g.50917023G>A | p.V759I | 0.51 | Germline |
| P58 |  |  |  |  |  |
| P59 |  |  |  |  |  |
| P60 |  |  |  |  |  |
| P61 |  |  |  |  |  |
| P62 |  |  |  |  |  |
| P63 | *BARD1* | 2:g.215610459C>A | p.E599D | 0.50 | Germline |
| P64 |  |  |  |  |  |
| P65 |  |  |  |  |  |
| P66 |  |  |  |  |  |
| P67 |  |  |  |  |  |
| P68 |  |  |  |  |  |
| P69 | *ATM* | 11:g.108138003T>C | p.F858L | 0.47 | Germline |
| P70 |  |  |  |  |  |
| P71 |  |  |  |  |  |
| P72 | *BRIP1* | 17:g.59924572G>A | p.R173C | 0.49 | Germline |
| P73 |  |  |  |  |  |
| P74 |  |  |  |  |  |
| P75 | *UIMC1* | 5:g.176395992C>G | p.R255T | 0.49 | Germline |
| P75 | *POLD1* | 19:g.50902726A>T | p.I101F | 0.5 | Germline |
| P76 |  |  |  |  |  |
| P77 |  |  |  |  |  |
| P78 |  |  |  |  |  |
| P81 | *RAD51* | 15:g.41023275G>C | p.G308R | 0.48 | Germline |
| P82 |  |  |  |  |  |
| P83 | *CHEK2* | 22:g.29121087A>G | p.I200T | 0.52 | Germline |
| P84 |  |  |  |  |  |
| P85 | *BARD1* | 2:g.215657152C>T | p.C78Y | 0.44 | Germline |
| P86 |  |  |  |  |  |
| P87 |  |  |  |  |  |
| P88 | *BRCA2* | 13:g.32911732del | p.C1081Vfs*6 | 0.48 | Germline |
| P88 | *ATM* | 11:g.108173586G>A | p.E1776K | 0.45 | Germline |
| P89 | *RAD54L* | 1:g.46738397C>T | p.P433L | 0.50 | Germline |

**Supplemental Table 7. IHC, Neoantigen and TCR Data**

**(Supplemental File 4 uploaded)**
